# Immunoproteasome in IgA Nephropathy: State-of-Art and Future Perspectives

**DOI:** 10.7150/ijbs.48330

**Published:** 2020-07-19

**Authors:** Ting Gan, Yang Li, Xu-jie Zhou, Hong Zhang

**Affiliations:** Renal Division, Peking University First Hospital; Peking University Institute of Nephrology; Key Laboratory of Renal Disease, Ministry of Health of China; Key Laboratory of Chronic Kidney Disease Prevention and Treatment (Peking University), Ministry of Education, Beijing, 100034, People's Republic of China; Research Units of Diagnosis and Treatment of Immune-mediated Kidney Diseases, Chinese Academy of Medical Sciences.

**Keywords:** IgA nephropathy, Glomerulonephritis, Immunoproteasome, Autoimmunity

## Abstract

IgA nephropathy (IgAN) is a leading cause of chronic kidney disease and renal failure. The exact pathogenesis of IgAN is not well defined, but some genetic studies have led to a novel discovery that the immunoproteasome probably plays an important role in IgAN. The immunoproteasome is a proteasome variant that is expressed when cells are stressed or receive inflammatory signals. While immunoproteasome is suggested to be mainly involved in major histocompatibility complex-I (MHC-I) antigen presentation, recent studies indicate that it may assert broad functions in trafficking events that activate both innate and adaptive immunity. In this review, we first summarize new insights into its functions in immunity, and discuss how it underlies its associations with IgAN. We also highlight its potential as a therapeutic target for the future.

## Introduction

IgA nephropathy (IgAN), the most common primary glomerulonephritis globally, is a leading cause of chronic kidney disease (CKD) and end stage kidney disease (ESKD) 1. Although the exact pathogenesis of IgAN is not well defined, a 'multi-hit' hypothesis has been proposed to explain the pathogenesis of IgAN. Specifically, the level of IgA1 bearing galactose-deficient O-glycans (Gd-IgA1) is increased in the circulation of patients with IgAN. These Gd-IgA1 are recognized as autoantigens by antiglycan autoantibodies, leading to the formation of immune complexes that accumulate in the glomerular mesangium 2. These immune complexes activate mesangial cells, induce proliferation and promote the production of extracellular matrix, cytokines and chemokines, leading to renal injury 3. However, this hypothesis does not explain the pathogenic mechanism of secondary IgAN. In addition, the etiologic factors in each hit remain largely unanswered. Newer insights arising from clinical and genetic studies contribute to the refinement of this hypothesis and suggest a genetic component to IgAN pathogenesis. It has been reported the familial aggregation of IgAN and the presence of Gd-IgA1 as a heritable trait 4. With significant improvements in genotyping efficiency and sequencing technology in recent years, several large genome-wide association studies (GWAS) for IgAN have been performed^4^-^8^, providing important insights into the potential mechanisms and pathways that contribute to the disease risk.

In one of the first large-scale GWAS conducted in IgAN, Gharavi, et al. 4 identified single nucleotide polymorphisms (SNP) located in the *PSMB8* locus (rs2071543, a Q49K missense variant) associated with the susceptibility to IgAN. The *PSMB8* locus encodes low molecular mass polypeptide 7 (LMP7) of the immunoproteasome, which is involved in antigen processing and presentation during infection. The typical exacerbation of hematuria in some patients following a mucosal infection suggests the participation of infectious agents in disease pathogenesis 9. In agreement with this notion, Coppo, et al. 10 reported an upregulation of the immunoproteasome in peripheral blood mononuclear cells (PBMC) of patients with IgAN. Furthermore, they observed a trend for an increased proteasome-immunoproteasome switch in patients with IgAN. It is of interest that the proteasome-immunoproteasome switch found in patients with IgAN was completely different from that of control subjects with respiratory or gastrointestinal infections without renal disease 9. And in a more recent study, the correlation between LMP7/β5 switch in peripheral white blood cells and velocity of progression of IgAN was reported, and high LMP7/β5 switch may represent a biomarker for identifying patients at risk of progression 11. These findings highlight the pathogenic significance of the proteasomal switch in IgAN.

In a shared genetics study between IgAN and systemic lupus erythematosus (SLE), we previously observed variants of *UBE2L3*, a gene involved in ubiquitin/proteasome pathway, were associated with susceptibility to both autoimmune diseases 12. *UBE2L3* has been further functionally validated involved in SLE by amplifying nuclear factor kappa B (NF-κB) activation and promoting plasma cell development 13. In a single-center open-label pilot trial testing the effect of bortezomib (a proteasome inhibitor that targets proteasome subunits β5, β5i and β1i) in patients with severe IgAN and significant proteinuria. Different to rituximab in other autoimmune disease, B-cell depletion in IgA nephropathy, which has been suggested to be an autoimmune disease, did not show expected efficacy 14. In comparison, after a 1-year follow-up in the trial, of all 8 subjects, 3 subjects (38%) had complete remission with proteinuria less than 300 mg per day 15. The evidence suggests that the (immuno) proteasome may play an important role in IgAN. And it supported that long term remission could be achieved without corticosteroid treatment but just by a short duration of bortezomib. In this review, we summarize the latest progress on (immuno) proteasome, discuss potential mechanisms of the (immuno) proteasome involved in the pathogenesis of IgAN, and suggest future research directions focused on (immuno) proteasome.

## Structure and activity of the (immuno) proteasome

**Figure 1:**
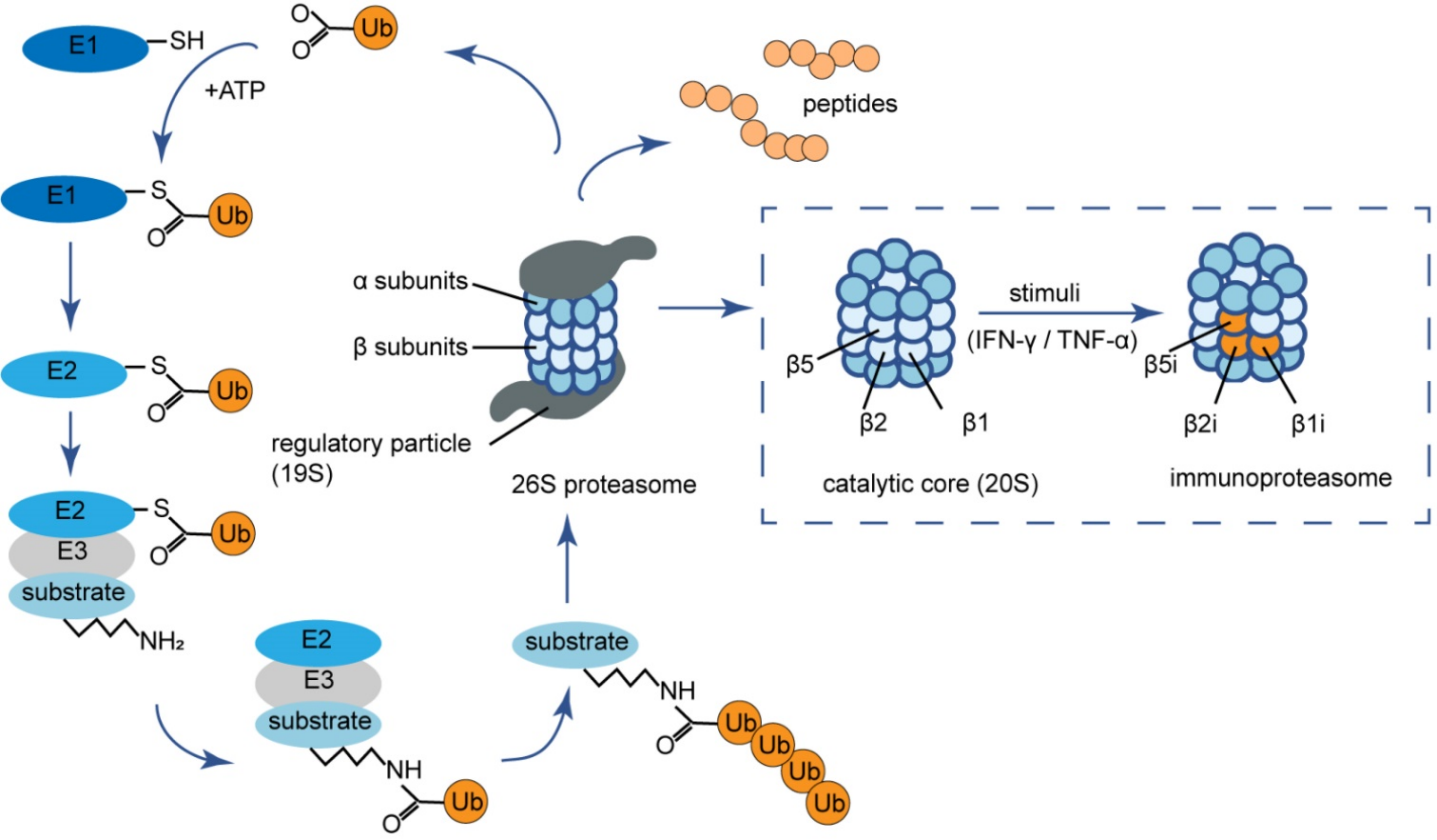
** The ubiquitin-proteasome system and (immuno) proteasome composition.** The ubiquitin-proteasome system is an ubiquitin-dependent proteolysis system. Substrates are first covalently attached to multiple ubiquitin moieties via the action of ubiquitin-activating enzymes (E1), ubiquitin-conjugating enzymes (E2) and ubiquitin ligases (E3) in an ATP-dependent manner. Poly-ubiquitinated proteins are then degraded by the 26S proteasome, which consists of a catalytic core complex, that is, the multi-subunit 20S proteasome (either constitutive or immunoproteasome) associated with two 19S regulator complexes governing the access to its catalytic cavity. The barrel shaped constitutive 20S proteasome is formed by four stacked rings of seven subunits each with the arrangement of α1-α7 or β1-β7. Under the stimuli of certain pro-inflammatory cytokines such as IFN-γ and TNF-α, the β1, β2, and β5 subunits are replaced by β1i (LMP2), β2i (MECL-1) and β5i (LMP7) respectively, forming the variant immunoproteasome. Ub: ubiquitin; ATP: adenosine triphosphate; IFN-γ: interferon-γ; TNF-α: tumor necrosis factor-α.

The 26S proteasome, the most common proteasome form in eukaryotic cells, is part of the ubiquitin-proteasome system (UPS) that is responsible for degrading damaged or misfolded proteins tagged with ubiquitin to maintain cellular homeostasis 16 (**Fig. 1**). The proteasome consists of two parts including 19S regulatory particles and a 20S catalytic core particle. The 20S proteasome is composed of four heptameric rings with a barrel-shaped structure. Seven distinct α-subunits (α1-α7) form the two outer α-rings and the two inner rings possess seven different β-subunits (β1-β7) 17. The proteolytic activity resides in β1, β2, and β5 subunits, which are responsible for the caspase-like (C-L), trypsin-like (T-L), and chymotrypsin-like (ChT-L) activities, respectively 18. Under the stimuli of certain pro-inflammatory cytokines such as interferon (IFN)-γ and tumor necrosis factor (TNF)-α, the β1, β2, and β5 subunits are replaced by functionally different counterparts named β1i (low molecular mass polypeptide 2, LMP2), β2i (multicatalytic endopeptidase complex‐like-1, MECL-1) and β5i (LMP7), forming the variant immunoproteasome 19. The immunoproteasome is predominantly expressed in lymphocytes and monocytes of jawed vertebrates 20. The changes in active site subunits cause an altered cleavage site preference as well as a different cleavage rate of immunoproteasome. In detail, the immunoproteasome has increased trypsin-like (T-L), and chymotrypsin-like (ChT-L) activities that are favorable for the production of antigenic peptides that bind to the groove of MHC-I 21. In fact, there are several hybrid immunoproteasomes with only one or two of the three immunoproteasome subunits integrated. Each hybrid proteasome has a unique structural composition as well as specialized and diversified enzymatic properties to produce a specific repertoire of peptides, thus regulates a host of cellular processes 22.

## Antigen presentation by immunoproteasome

From a clinical point of view, about 30% of patients with IgAN (mostly young subjects) have a clear history of disease exacerbation after upper respiratory or gastrointestinal infections 23. Thus, IgAN has traditionally been considered as correlated to mucosal infections. Mucosal immunity is triggered by recognition of bacterial or viral nucleic acids or products or other pathogen-associated molecular patterns (PAMPs) by toll-like receptors (TLRs) 24. The TLR activation triggers a cascade of intracellular messages leading to activation of the interferon regulatory factor (IRF) and the NF-κB. The Stat-1/IRF-1 dimers are the major transcription factors involved in IFN-γ signal transduction. Under the stimuli of IFN-γ, the expression level of immunoproteasome subunits was significantly upregulated. This cytokine-induced expression is the result of binding Stat-1 and IRF-1 transcription factors to multiple IFN-γ consensus/activation sequences in the promoter region of the *LMP2*, *LMP7*, and *MECL-1* genes 25. In agreement, it has been reported that genes involved in the immunoproteasome pathway were activated in IgAN during phases of clinical activity 10. Coppo, et al. 10 observed upregulated expression of the immunoproteasome subunits mRNA in PBMC from patients with IgAN. However, precise molecular mechanism of the upregulation of immunoproteasome and how it is involved in pathogenesis and/or progression of IgAN remain obscure.

Recent studies show both pathogenic and prognostic role of anti-glycan antibodies in IgAN, suggesting that IgAN is a type of autoimmune disease 26-28. Aberrantly glycosylated IgA1 might be recognized as an autoantigen, and thus the immune response against it may lead to the production of anti-IgA1-antibodies. However, of interest, more recent studies suggest that some pathogens such as Epstein-Barr virus and Streptococcus bacteria possess GalNAc-containing structures on their surfaces, which might subsequently cross-react with glycans on IgA1 resulting in the formation of pathological immune complexes 29-31. This 'molecular mimicry' might explain the association of macroscopic hematuria with upper respiratory tract infections 23. During intracellular viral or bacterial infection period, it is supposed that enhanced immunoproteasome activation in IgAN may lead to an increased presentation of self-antigens onto MHC-I molecules, ultimately resulting in enhanced autoimmune responses. In this process, the immunoproteasome may cleave either endogenous or exogenous proteins to generate antigen peptides, which are firstly complexed to MHC-I in the endoplasmic reticulum and then exposed on the plasma membrane of antigen-presenting cells (APCs), for either direct or cross-presentation to CD8+ T-lymphocytes^21^. Owing to unique structural features and proteolytic activity of the immunoproteasome, the process is accomplished at a higher rate and with greater efficacy compared with constitutive proteasome.

## Cytokine production facilitation

**Figure 2:**
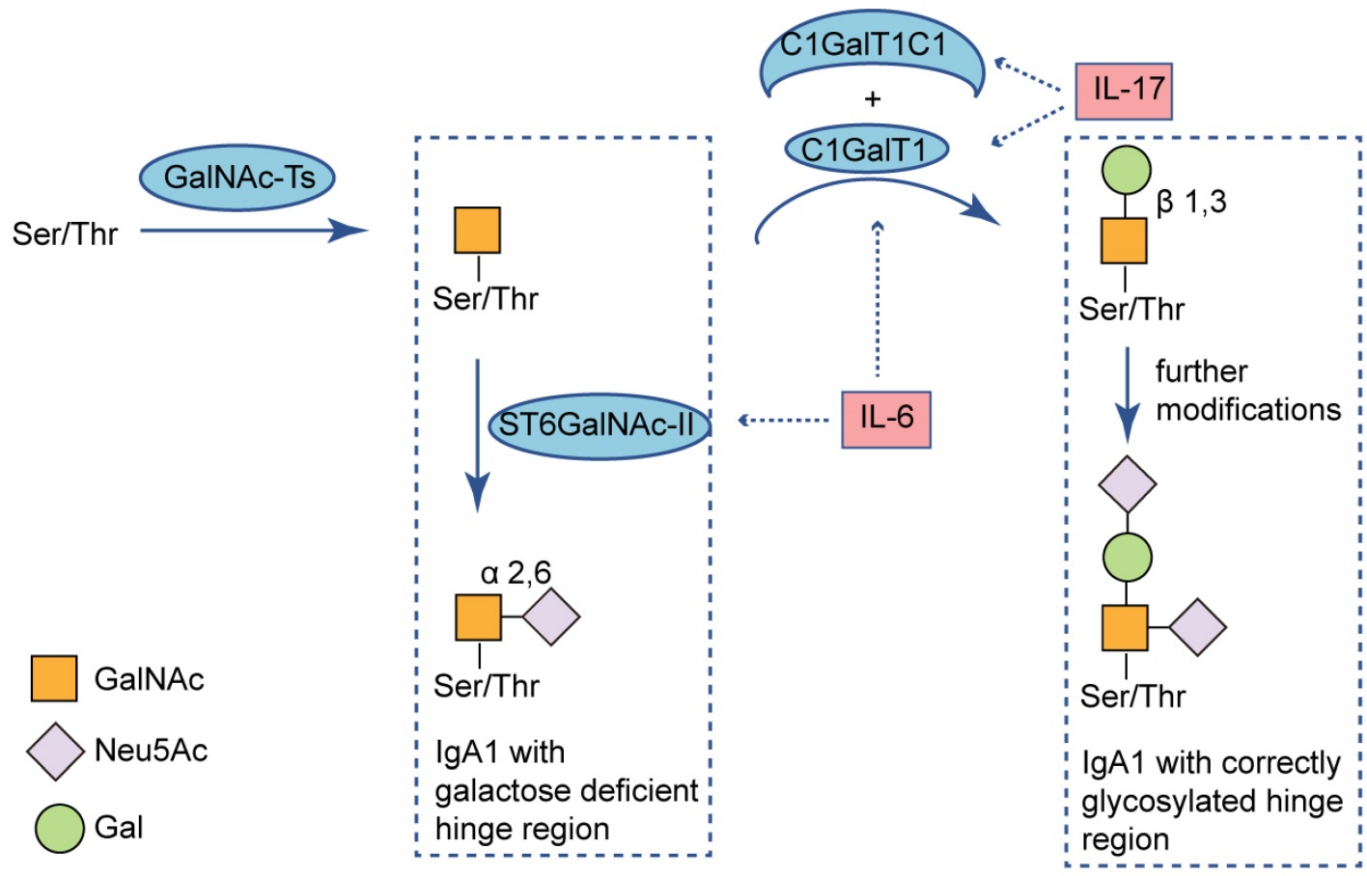
** Immunoproteasome-related cytokines alter IgA1 O-glycosylation.** O-glycans are synthesized in a stepwise manner, starting with attachment of N-acetylgalactosamine to Ser/Thr residues of the hinge region (catalyzed by GalNAc-Ts), followed by addition of galactose (catalyzed by C1GalT1 and its chaperone C1GalT1C1). Sialic acid can be added to each glycan by ST6GalNAC-II for attachment to GalNAc and this can prevent subsequent addition of galactose. IL-17 decreases expression of C1GalT1 and its chaperon C1GALT1C1; IL-6 increases expression of ST6GalNAC-II and decreases expression of C1GalT1. Both of the immunoproteasome-related cytokines can stimulate production of Gd-IgA1. Ser: serine; Thr: threonine; GalNAc: N-acetylgalactosaminyl; Neu5Ac: N-acetylneuraminic acid; Gal: galactose; GalNAc-Ts: N-acetylgalactosaminyl-transferases; ST6GalNAc-II: α-*N*-acetylgalactosaminide α-2,6-sialyltransferase 2; C1GalT1: core 1 β1,3-galactosyltransferase; C1GalT1C1: C1GalT1 chaperone 1; IL-17: interleukin-17; IL-6: interleukin-6.

As mentioned above, the TLR activation triggers a cascade of intracellular messages leading to activation of the NF-κB transcription pathway 24. Coppo, et al. 10 noticed that nuclear translocation of NF-κB p50 and p65 were significantly enhanced in patients with IgAN compared to healthy controls. As known, activation of NF-κB leads to release of cytokines and chemokines and killing pathogens via the oxidative burst 24. Also, cytokines might participate in an aberrant IgA1 galactosylation. For example, IL-17 downregulates the expression of C1GalT1 and C1GalT1 specific chaperone 1 (C1GalT1C1), leading to higher secretion of Gd-IgA1 from B cells 32. And IL-6, promotes hypogalactosylation of IgA1 by increasing ST6GalNAc-Ⅱ activity and decreasing activity of C1GalT1 33 (**Fig. 2**). Recent studies have elucidated roles of the immunoproteasome beyond antigen presentation. The enhanced immunoproteasome activation in IgAN may be associated with regulating inflammatory pathways, through a direct effect on NF-κB signaling and the production of proinflammatory cytokines 25, 34, 35.

NF-κB is a pivotal transcription factor that promotes growth factors, proinflammatory cytokines, and cell survival signaling. Evidence from immunoproteasome-deficient mice has provided compelling support for the role of immunoproteasome in NF-κB activation. It was observed that cell lines lacking LMP2 had defects in processing NF-κB precursors (p100/p105) to the active transcription factors (p52/p50) 25. Furthermore, in preclinical animal models, selective immunoproteasome inhibitors were found to reduce the secretion of proinflammatory TNF-α, IL-6, and IL-23 through altering NF-κB activity 22. Several mutations in the human *PSMB8* and *PSMB9* genes encoding the LMP7 and LMP2 immunoproteasome subunits, respectively, have been discovered in autoinflammatory disorders 17, 36. In cells from patients with *PSMB8*-associated mutations in LMP7, basal IL-6 production was significantly higher compared to healthy controls 36. These examples show that the immunoproteasome content can affect cytokine production. And compared with the constitutive proteasome (nearly 82 minutes), the decreased time in which immunoproteasome can be formed (only 21 minutes) makes the immunoproteasome provide a rapid response to inflammation or stress as signaled by proinflammatory cytokines 22.

## T cell differentiation

As an immunological disorder, accumulating research evidence supports the involvement of multiple abnormal lymphocyte subsets in IgAN. Although a heterogeneous disease, a report observed higher proportions of circulatory Th2 and Th17 cells, lower Th1 and Treg cells in patients with IgAN 31. Recent studies show that immunoproteasome activity promotes helper T (Th) cell differentiation (including proinflammatory Th1 and Th17 cells) and effector T cell expansion (cytotoxic CD8 cells), while repressing regulatory T (Treg) cell induction 22. And the immunoproteasome subunit LMP7, coded by *PSMB8* gene, is necessary for Th17 differentiation and inhibits Treg differentiation 37. It was found that PBMC of IgAN patients had higher expression of *PSMB8* gene, especially those individuals with high proteinuria 9. And GWAS reported protective genotypes in IgAN showed lower *PSMB8* gene expression 3. Thus, the immunoproteasome has been proposed to perform specialized roles in T cell differentiation and it may participate in pathogenesis and pathophysiology of IgAN 37. Enhanced immunoproteasome activation may contribute to Th17/Treg disequilibrium in IgAN. Apart from participation in the immune response against infection, cytokines like IL-17 can also impact aberrant IgA1 galactosylation in IgAN 33. In IgAN, higher proportions of Th17 lymphocytes secrete high concentrations of IL-17, which can stimulate production of Gd-IgA1. However, Treg cells cannot effectively suppress the defective immune response leading to formation of immune complexes. All these processes can cause formation and deposition of circulating immune complexes in glomeruli 31. In agreement, Lin, et al. 38 demonstrated that IgAN patients had an imbalance of Treg/Th17 and in particular, the altered distribution of Treg and Th17 in circulation and local renal tissues were correlated with prognostic parameters of IgAN such as impaired glomerular filtration rate (GFR), proteinuria, high blood pressure and tubulointerstitial injury.

## B cell immunity

**Figure 3:**
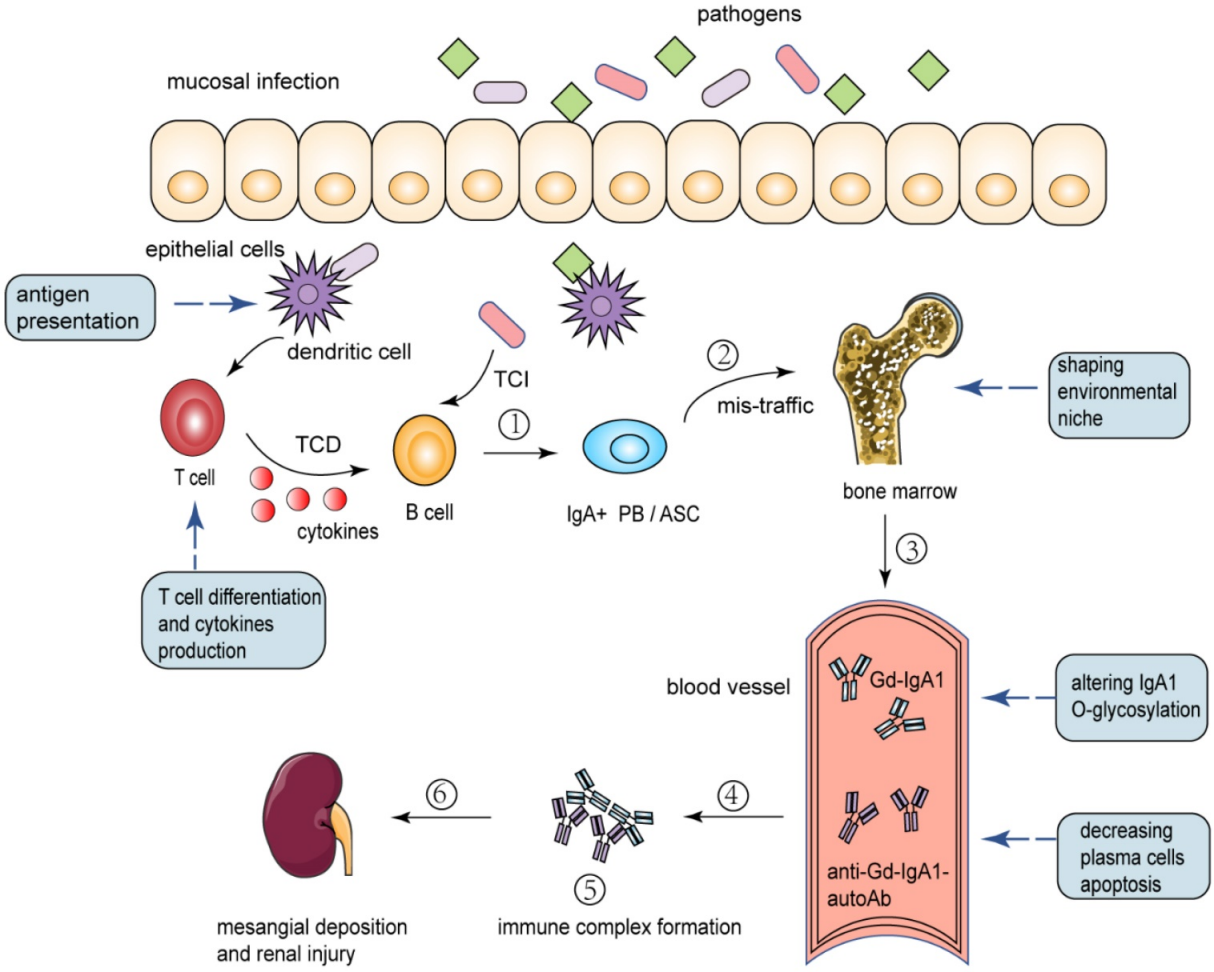
** Proposed pathways the immunoproteasome involved in the pathogenesis of IgAN.** (1) Mucosal infection primes naive B cells to class switch to become IgA^+^ plasmablasts (PB) and antibody-secreting cells (ASC) through T-cell-dependent (TCD) and T-cell-independent (TCI) processes. Antigen presentation by immunoproteasome promotes immune response, T cell differentiation and cytokines production. (2) A proportion of antigen-committed IgA PB and ASC with altered homing receptors and therefore they mistakenly “home” to the bone marrow. Immunoproteasome-related cytokines can contribute to the environmental niche in bone marrow. (3) These translocated cells take up residence in the bone marrow, where they secrete IgA into the systemic circulation. Factors such as genetic background and immunoproteasome-related cytokines can alter IgA1 O-glycosylation and lead to Gd-IgA1 production. (4) Plasma cells secrete IgG and IgA autoantibodies directed against the Gd-IgA1 hinge region O-glycans. In the phase of intense secretion of antibodies, immunoproteasome can balance the unfavorable proteasomal load/capacity ratio and decrease plasma cells apoptosis. (5) Formation of immune complexes from autoantigen (Gd-IgA1) and O-glycan-specific antibodies. (6) Deposition of pathogenic immune complexes in the mesangium, activation of mesangial cells, and induction of glomerular injury. PB: plasmablasts; ASC: antibody-secreting cells; TCD: T-cell-dependent; TCI: T-cell-independent; Gd-IgA1: galactose-deficient IgA1; Anti-Gd-IgA1-autoAb: Anti-Gd-IgA1-autoantibody.

In IgAN, some data suggest bacterial and viral antigens from antigens involved in upper respiratory and gastrointestinal mucosal infections could be found in the glomeruli in association with IgA deposits 23. After encountering an antigen, B-lymphocytes activate a complex program involving proliferation, generation of memory cells, isotype switch, and affinity maturation 39. Ultimately, B cells differentiate into plasma cells (PC) and produce antibodies to control and eliminate infection. The increased serum levels of Gd-IgA1, glycan-specific IgA and IgG autoantibodies in IgAN patients were produced by plasma cells (**Fig. 3**). Although some of them home in the bone marrow and survive for longer periods, most plasma cells are short-lived after a few days of intense immunoglobulin (Ig) secretion 39. Research has demonstrated that B cells require complex regulation of proteasome activity during differentiation and secretion of antibodies 40. The limiting plasma cell lifespan may be caused by a dramatic decrease of the relative amount and proteolytic activity of proteasome in the late phases of plasmacytic differentiation.

In general, the proteasome can degrade endoplasmic reticulum (ER) proteins which failed to fold properly 41, and this termed as ER-associated degradation (ERAD). Moreover, the proteasome plays an important role in unfolded protein response (UPR). The signaling pathway called UPR ensures that plasma cells can handle the proper folding of Ig proteins. When the ER transmembrane endoribonuclease and kinase called IRE1 senses unfolded proteins, it oligomerizes and is activated by autophosphorylation, thus allowing it to excise an intron of the long isoform of X-box binding protein 1 (XBP-1μ), creating a short isoform of XBP-1 (XBP-1s) 42. The XBP-1μ protein, which could be degraded through the ubiquitin-proteasome pathway, can act as a dominant-negative inhibitor of XBP-1s activity 43. In addition to quieting ER stress by activating the UPR, XBP-1s is also a transcription factor known to be selectively and specifically required for the terminal differentiation of B lymphocytes to plasma cells 44. However, it was surprising to observe that the enzymatic activity and the relative abundance of proteasome decreased during plasma cell differentiation 39. With progressive fall of proteolytic capacity, accumulated ERAD substrates and XBP-1μ protein and suppressed XBP-1s activity together lead to ER stress and cell apoptosis. Thus, the apoptosis of plasma cell was promoted by an unbalance between load (immunoglobulin synthesis) and capacity of the proteolytic machinery (proteasome).

It is speculated that the upregulation of the immunoproteasome in IgAN may partly balance the unfavorable proteasomal load/capacity ratio and prolong plasma cells survival. Similar to the constitutive proteasome, the immunoproteasome can also degrade ERAD substrates, misfolded and or damaged proteins to limit the accumulation of unwanted proteins and decrease cell apoptosis 45. On the other hand, it has been reported that immunoproteasome-deficient mice have altered NF-κB activity, leading to compromised B cell numbers, survival, and function 34. Apart from this, there is increasing recognition for the presence of a mucosa-bone marrow axis in the pathogenesis of IgAN 46. Large numbers of polymeric IgA1-positive plasma cells are found in the bone marrow (BM) in IgAN and these long-lived plasma cells may maintain a high level of IgA1 in the blood 47. It is clear that extrinsic factors such as cytokines play important roles in shaping the environmental niche that promotes survival of PC in the BM. Intriguingly, at least two key cytokines contributing to the plasma cell niche, TNF and IL-6, have been demonstrated to be dependent upon the immunoproteasome subunit LMP7 48. In agreement with this notion, Li, et al. 49 reported that immunoproteasome inhibitor ONX 0914 can induce plasma cell apoptosis through activating the UPR and suppressing plasma cell survival factors in the bone marrow.

## The immunoproteasome as a therapeutic target

In recent years, studies have elucidated roles of the immunoproteasome beyond antigen presentation. The immunoproteasome participates in a variety of biological processes, including protein quality control, transcription, immune response, cell signaling, and apoptosis and so on 50. The immunoproteasome dysfunction or dysregulation, therefore, is closely related to a wide range of diseases such as tumor, infectious diseases, central nervous system diseases, autoimmune and inflammatory diseases 16. As a result, significant research has focused on elucidating the role of the immunoproteasome in a variety of diseases to evaluate its potential as a therapeutic target. Indeed, the proteasome inhibitor bortezomib has long been on the market as an Food and Drug Administration (FDA)‐approved therapy for multiple myelomas, and many other proteasome inhibitors are being investigated 51. However, most of these inhibitors alter the catalytic activity of both the constitutive proteasome and the immunoproteasome indiscriminately, leading to various unwanted side effects and the application has been limited 52. To overcome these off-target effects, efforts are being made to design selective inhibitors that specifically target immunoproteasome subunits. The immunoproteasome-specific therapy can selectively affect the function of activated immune cells while sparing other cell types that would be damaged by bortezomib 53. In preclinical animal models, selective immunoproteasome inhibitors are beneficial to the treatment of many different diseases including hematologic malignancies, experimental colitis, experimental arthritis, systemic lupus erythematosus, and multiple sclerosis 48, 54-57. These may be because the immunoproteasome inhibitors affected a multitude of cellular responses such as endoplasmatic reticulum stress, unfolded protein response, NF-κB inhibition, cytokine modulation, T cell differentiation, or an increase in proapoptotic factors and tumor suppressors 58. In addition to the diseases in which the level of the immunoproteasome expression or activity is higher than normal, there are also different diseases in which the immunoproteasome is hindered, which may benefit from the stimulation of immunoproteasome activity. One possible class of diseases that could benefit from immunoproteasome stimulation is viral infections 16.

Notably, several interesting trials testing the effect of proteasome inhibitor in patients with IgAN and significant proteinuria have been performed 15, 59. The outcomes of a single-center open-label pilot trial showed the potential of therapy targeted at (immuno) proteasome in IgAN treatment. After 1-year follow-up, of all 8 subjects who received and tolerated 4 doses of bortezomib over a 2-week period during enrollment, 3 subjects (38%) had complete remission defined as proteinuria of less than 300 mg per day 15. However, the trial has several significant limitations, including small sample size, no control group and nonrandomization. Besides, bortezomib targets proteasome subunits β5, β5i and β1i, and alters the catalytic activity of constitutive proteasome and immunoproteasome indiscriminately. Thus, more evidence is needed to verify the conclusion of this trial and the efficacy of (immuno) proteasome inhibition in IgAN. As an autoimmune disease elicited by Gd-IgA1 antibodies, immunoproteasome inhibitors may be considered as a promising strategy due to its important role in plasma cells survival 49. However, before the immunoproteasome becomes a therapeutic target in IgAN, further research is necessary to clarify whether and how the immunoproteasome participate in pathogenesis and/or progression of the disease. The advantages and disadvantages of immunoproteasome as a therapeutic target are summarized in **Table 1** 60-67.

## Future directions

In this review, we discuss the important role of immunoproteasome in several mechanisms respectively and it is likely that these mechanisms synergistically contribute to the susceptibility to IgAN and risk of disease progression. The dysfunction or dysregulation of the immunoproteasome may play a crucial role in IgAN pathogenesis and/or progression **(Fig. 3).** However, clinical implications of the immunoproteasome in IgAN are still largely unexplored. There is much to be done in this area. For instance, areas of interest might include the following: (i) genetic analysis of the variation of immunoproteasome components and their correlation with immunoproteasome function; (ii) determining whether and how immunoproteasome impact the pathogenesis of IgAN; (iii) development of IgAN animal models that harbor mutations in the immunoproteasome gene; (iv) determining whether immunoproteasome can serve as a biomarker for clinical activity and aid in genetic screening of IgAN susceptible groups; (v) studying the potential of immunoproteasome as a therapeutic target in IgAN. Elucidating the exact pathological contribution of immunoproteasome dysfunction or dysregulation to the development of IgAN and the underlying causal mechanisms will undoubtedly bring valuable insights into the pathogenesis of IgAN and lead to the development of novel therapeutic approaches.

## Figures and Tables

**Table 1:** Advantages and disadvantages of immunoproteasome as a therapeutic target

Advantages	Descriptions
Less off-target effects	The immunoproteasome-specific therapy targets subunits of immunoproteasome
Reducing the toxic and side effects	The immunoproteasome-specific therapy selectively affects the function of activated immune cells while sparing other cell types
Efficient immune regulation	The immunoproteasome participates in a variety of immune processes
Broad biological applications	Recent studies confirm that the immunoproteasome is related to many human diseases such as autoimmune diseases, central nervous system diseases and hematologic malignancies^60^-^63^
Less drug resistance	The potential of selective immunoproteasome inhibitors in overcoming drug resistance has been reported^64^, ^65^
**Disadvantages**	**Descriptions**
Further basic research is needed	More basic studies on immunoproteasome structure and functions are needed to guide immunoproteasome-specific therapy^66^, ^67^
Influence on some physiological processes	Apart from immune response, immunoproteasome is involved in some physiological processes such as protein degradation, cell proliferation and survival

## Abbreviations

IgAN: IgA nephropathy
MHC-I: major histocompatibility complex-I
CKD: chronic kidney disease
ESKD: end stage kidney disease
Gd-IgA1: galactose-deficient IgA1
GWAS: genome-wide association studies
SNP: single nucleotide polymorphisms
LMP: low molecular mass polypeptide
PBMC: peripheral blood mononuclear cells
SLE: systemic lupus erythematosus
NF-κB: nuclear factor kappa B
UPS: ubiquitin-proteasome system
IFN: interferon
TNF: tumor necrosis factor
MECL-1: multicatalytic endopeptidase complex‐like-1
PAMPs: pathogen-associated molecular patterns
TLRs: toll-like receptors
IRF: interferon regulatory factor
APCs: antigen-presenting cells
IL-17: interleukin-17
C1GalT1C1: C1GalT1 specific chaperone 1
Th cell: helper T cell
Treg cell: regulatory T cell
GFR: glomerular filtration rate
PC: plasma cells
Ig: immunoglobulin
ER: endoplasmic reticulum
ERAD: endoplasmic reticulum associated degradation
UPR: unfolded protein response
XBP-1: X-box binding protein 1
BM: bone marrow
FDA: Food and Drug Administration
